# Report of Deaths in the City of Buffalo for the Month of October, 1862

**Published:** 1863-01

**Authors:** Sandford Eastman

**Affiliations:** Health Physician


*Report of Deaths in the City of Buffalo for the month of October*, 1862.

Accident 9, do by drowning 3, Apoplexy 2, Bronchitis 1, Cancer 1, do stomach 1, do womb 1, Consumption 14, Convulsions 6, Croup 9, Debility 1, Delirium tremens 4, Diarrhœa 1, Disease of the brain 1. do heart 2, do liver 1, do kidney 1, do spine 1, Diptheria 4, Dropsy 5, Dysentery 2, Erysipelas 2, Fever 4, do puerperal 2, do scarlet 7, do typhoid 8, Gun-shot wound 1, Hæmorrhage from lungs 1, do uterus 1, Inflammation of the bowels 1, do brain 4, do and meninges 6, do lungs 7, do do typhoid 1, do peritoneum 1, do stomach 1, do womb 1, Insanity 4, Old age 6, Paralysis 1, Premature birth 1, Pyaemia 1, Scrofula 1, Suicide 1, Tetanus 1, Thrush, infantile 1, Unknown 3. Total, 139.

SANDFORD EASTMAN, M. D., Health Physician.

